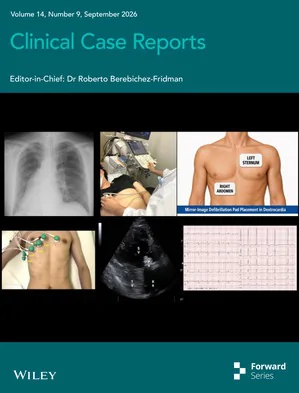# Cover Image

**DOI:** 10.1002/ccr3.73409

**Published:** 2026-08-28

**Authors:** Junya Shimamoto, Rintaro Tamaruya

## Abstract

The cover image is based on the article *A Unified Three‐Step Mirror‐Image Protocol for ECG, Echocardiography, and Cardioversion in Dextrocardia: A Case Report* by Junya Shimamoto and Rintaro Tamaruya, https://doi.org/10.1002/ccr3.73122.